# Phylodynamics analysis of HIV epidemic history in Belarus in 1987–2022

**DOI:** 10.3389/fepid.2025.1601976

**Published:** 2025-07-21

**Authors:** Alexander Kirpich, Alina Nemira, Ayotomiwa E. Adeniyi, Aleksandr Shishkin, Anastasia S. Bunas, Natalya D. Kolomiets, Irina N. Glinskaya, Yuriy Gankin, Elena L. Gasich, Pavel Skums

**Affiliations:** ^1^ Department of Population Health Sciences, Georgia State University, Atlanta, GA, United States; ^2^ Department of Computer Science, Georgia State University, Atlanta, GA, United States; ^3^ Laboratory for the Diagnosis of HIV and Opportunistic Infections, Center for Hygiene, Epidemiology, and Public Health, Minsk, Belarus; ^4^ Department of Clinical Microbiology, Diagnostics, and Epidemiology, Belarusian State Medical University, Minsk, Belarus; ^5^ Division of HIV Infection and Parenteral Viral Hepatitis Prevention, Center for Hygiene, Epidemiology, and Public Health, Minsk, Belarus; ^6^ Quantori, Cambridge, MA, United States; ^7^ School of Computing, University of Connecticut, Storrs, CT, United States

**Keywords:** Belarus, HIV, phylodynamics, molecular epidemiology, infectious diseases

## Abstract

This paper presents the first systematic molecular epidemiology study of the HIV epidemic in Belarus, an Eastern European country that, like much of Eastern Europe and including the Post-Soviet region, has been largely understudied in relation to HIV epidemics. HIV sequences collected nationwide between January 2018 and May 2022 were analyzed using phylogenetic and phylodynamic methods. The findings reveal two distinct epidemic waves spanning 1997–2005 and 2009–2018, each driven by different dominant modes of transmission. The study also identifies potential introductions and intra-country transmission routes, emphasizing the pivotal role of the capital city and eastern industrial hubs within Belarus in shaping the epidemic’s trajectory. This work addresses an important gap in understanding HIV dynamics in Eastern Europe.

## 1 Introduction

The global Acquired Immunodeficiency Syndrome (AIDS) pandemic, caused by the Human Immunodeficiency Virus (HIV) and first documented in 1981, remains a major public health challenge. Despite substantial efforts to combat HIV, the Joint United Nations Programme on HIV/AIDS (UNAIDS) estimated that by the end of 2023, 36.1–44.6 million people were living with HIV worldwide (1), and 650,000 people died from AIDS-related illnesses in 2021 (1).

Although antiretroviral therapy and preventive measures have reduced new infections in many regions (2), progress has been uneven. In particular, Eastern Europe is one of the few regions where both new HIV infections and AIDS-related deaths continue to rise (3). Along with Sub-Saharan Africa, it has one of the highest HIV incidence rates globally (3), leaving it off track to meet the 2030 AIDS elimination targets (4). Within Eastern Europe, former Soviet Union (FSU) countries account for 92% of all newly registered HIV cases (4)

Despite these concerning trends, Eastern Europe in general and FSU countries in particular remain relatively neglected in HIV epidemiological research. Among the major regions analyzed in a recent global burden of HIV study (3), it has the fewest PubMed-indexed publications—approximately 19 times fewer than Sub-Saharan Africa and 13 times fewer than North America.

One of FSU countries is the Republic of Belarus, which has a population of 9.155 million (5). Like many nations worldwide, it faces multifaceted challenges associated with HIV pandemic. The country has experienced a steady annual increase in both registered HIV cases and incidence rates, with the latter rising from 9.9 per 100,000 in 1996 to 25.9 per 100,000 in 2017.

HIV infection was first identified in Belarus in 1987, with only 113 recorded cases by 1995, primarily among individuals who had arrived in the country for work or study from abroad (6). However, the epidemiological situation changed dramatically in 1996 with the discovery of a localized outbreak in the southeastern cities of Svietlahorsk and Zlobin. Over six months, 1,021 new cases were registered, 954 (93%) of which were linked to injection drug use (7, 8). Consequently, the incidence rate surged from 0.07 per 100,000 in 1995 to 9.9 per 100,000 in 1996.

Following this outbreak, HIV spread rapidly across the country (9–14). From 1996 to approximately 2004, parenteral transmission i.e., via injection accounted for the majority of new cases, comprising 91.5% of infections in 1996 (15). After 2004, sexual transmission—predominantly through heterosexual contact—became the dominant mode of new infections (12, 16, 17). By 2023, the proportion of parenteral transmission via injection drug use had declined to 14.4%, while sexual transmission accounted for 83.2% of new cases (11, 18, 19).

The only exception in this trend was the mid-2010s period, when the number of new HIV cases has increased from 1,533 cases in 2013 to 2,468 cases in 2017. This has been attributed to the intensification of parenteral transmission routes, and changes in the drug scene. Since 2019, a decrease in the number of newly identified HIV infections has been observed, with the exception of 2022, when the number of newly diagnosed cases slightly increased by 11.3% compared to 2021, which may be attributed to the delayed detection of individuals who were not properly diagnosed during 2020–2021 due to the COVID-19 pandemic—the phenomenon observed in several countries (20–22).

Currently, the epidemiological situation regarding HIV infection in Belarus is classified as a concentrated stage of the HIV epidemic, in accordance with internationally accepted criteria (17, 23). The number of people living with HIV (PLHIV) as of January 1, 2024, was 25,038, with an estimated total of 27,000 PLHIV for the same year (24).

In general, the HIV epidemic in Belarus remains concentrated among high-risk groups, including people who inject drugs (PWID), men who have sex with men (MSM), sex workers (SW), and people in detention (PID). Other transmission modes play a relatively minor role; for example, as of January 1, 2018, the vertical transmission rate (mother-to-child) was 1.3% (25, 26).

While traditional epidemiological statistics provide valuable insights, understanding HIV transmission dynamics can be greatly enhanced through molecular epidemiology. In recent decades, particularly during the 2010s, advances in molecular techniques, decreasing sequencing costs (27, 28), and improvements in computational methodologies (29) have established phylogenetic and phylodynamic analyses as essential tools in epidemiological investigations (30). This is especially relevant for HIV, which exhibits extreme genomic heterogeneity due to its rapid replication and the high error rate of reverse transcription (31–36).

Previous molecular epidemiology studies of HIV in Belarus have focused on assessing within-country HIV genomic diversity. These studies identified the A6 sub-subtype of HIV-1 as the dominant lineage circulating in Belarus (37), consistent with trends observed across FSU countries (38–40). Initially, A6 was primarily transmitted among PWID, but over time, heterosexual transmission became the predominant mode of spread (38, 41–43). Additionally, isolated cases of recombinant forms CRF03_AB and CRF02_AG have been reported (42, 44–46).

However, the sequencing data collected by Belarusian epidemiologists over the past decade, combined with existing phylodynamic methodologies, enables more detailed and higher-resolution analysis of the HIV epidemic. These approaches allow for the inference of key epidemiological parameters, transmission routes, and geographical distribution (47).

This study aims to conduct a comprehensive phylogenetic and phylodynamic analysis of HIV epidemiological dynamics in Belarus. Similar studies have been performed in Uzbekistan (48), neighboring Ukraine (49), Poland (50), Russia (51), and more broadly across FSU countries (52–55), as well as in other regions (56–58). However, without epidemiological data from Belarus, the understanding of HIV-1 transmission dynamics in the FSU region and neighboring areas remains incomplete. This analysis will help fill this gap and provide new insights into the molecular epidemiology of HIV in Eastern Europe.

## 2 Methods

### 2.1 Data generation and pre-processing

Blood samples were collected by the Republican Center for Hygiene, Epidemiology, and Public Health (RCHEPH) in Minsk, Belarus, between 2018 and May 2022 as part of routine HIV screening and monitoring. Sample processing and sequencing were conducted by the Laboratory for HIV and Opportunistic Infections Diagnosis. The study included both antiretroviral treatment—experienced and treatment-naïve individuals. The geographical distribution of sequences collection regions generally correlates with officially reported HIV prevalence (Pearson’s r=0.79, comparing sequence counts and regional prevalence rates).

Viral RNA was extracted from 100 μl of blood plasma using Ribo-Prep following the manufacturer’s instructions. Sequencing was performed on a 3,500 Genetic Analyzer (Applied Biosystems, USA), with base calling conducted using the manufacturer-supplied sequencing analysis software.

For hosts with multiple samples, the sample with the median collection date was retained for further analysis. This resulted in a dataset of 867 HIV pol gene amplicons. The amplified HIV-1 pol fragment used for the analysis covered positions 4,177–5,211 in relation to HXB2 genome reference. The resulting sequences were assembled and manually edited using Sequencing Analysis Software v6.0, SeqScape® Software v3.0 (Applied Biosystems), and Geneious® v8.0 (Biomatters Ltd.). The sequences and associated patient metadata (collection date, place of residence) are publicly available via GenBank (accession numbers OP330328–OP331194).

Nucleotide sequence alignment was carried out using Clustal W (59) and MAFFT (60). Groups of individual sequences were processed using BioEdit v6.1 and Geneious® v8.0. The alignment was refined by generating a consensus sequence with EMBOSS Cons (61), realigning sequences to the consensus, and removing those with excessive gaps or poor alignment quality. HIV-1 subtyping was performed using COMET HIV-1 (62) and REGA (63), with sequences with undetermined subtypes further analyzed for recombination using SimPlot (64). After data cleaning and duplicate removal, the final dataset consisted of 640 sequences of subtype A/A6, each 1,032 bp in length, which was then used for further analysis.

All data used in the analysis were depersonalized, and ethical approval was obtained from RCHEPH. Additionally, the study was designated as not human subjects research by the Georgia State University Institutional Review Board (GSU IRB Number: H25158).

### 2.2 Inference and analysis of HIV introduction pathways and transmission clusters

To infer potential HIV introductions into Belarus, the study dataset was expanded by incorporating globally collected subtype A pol gene sequences downloaded comprehensively from the Los Alamos National Laboratory (LANL) HIV sequence database.

The combined dataset was analyzed using the Nextstrain phylogenetic workflow (65) implemented in augur (version 24.3.0) (66). This included data primary processing (sequence indexing, filtering, and multiple sequence re-alignment), followed by phylogenetic analysis. This second step proceeded in two main stages: first, a maximum likelihood phylogenetic tree was constructed from the aligned sequences using RAxML (67). This initial tree was then refined with TreeTime (68) to generate a time-resolved phylogeny and infer ancestral timing.

Finally, the annotated phylogenetic tree, with leaf traits representing the geographical locations of sequence collection, was analyzed using PastML (69). Ancestral traits representing most likely geographical locations of ancestral HIV variants were reconstructed via maximum likelihood inference using the F81 model of trait evolution (70). The inferred ancestral traits of the most recent common ancestors of intra-Belarusian lineages were interpreted as the likely sources of HIV introduction into the country.

To reconstruct and visualize within-country transmission clusters and potential transmission networks, we used MicrobeTrace (71). This tool builds a genetic relatedness network by connecting sequences whose pairwise genetic distance falls below a predefined threshold. The resulting network serves as an approximation of the underlying transmission network (72). Given the extended temporal and geographic span of our dataset, we used a 2.5% distance threshold, consistent with prior studies of large transmission clusters spanning long time periods (56, 73, 74).

To assess the contribution of local and generalized transmissions, the assortativity coefficient of inferred network with respect to city and region of host residence was calculated. The coefficient measures the tendency of inferred transmission links to connect hosts with the same geolocation traits. For the calculations, we used the script from (75, 76) with Python v3.10.0 and networkx library.

Both external and intra-country transmission networks were visualized using Inkscape v0.92. Base maps were obtained from IGISMAP and SimpleMaps. Transmission links generated by MicrobeTrace were integrated with geolocation data using ArcMap v10.7.

### 2.3 Phylodynamic analysis

All phylodynamic analyses were conducted using BEAST 2.6.7 (77) with BEAGLE 4.0.0 (78) for computational acceleration. First, temporal signal was evaluated by by regressing root-to-tip genetic divergence against sampling dates using TempEst (79). Next, a non-parametric Bayesian skyline plot (BSP) model (80) was used to reconstruct the HIV phylogeny and estimate the dynamics of the effective population size of the infected population. We employed the GTR nucleotide substitution model, a relaxed molecular clock, and a piecewise constant effective population size ($N_{e}$) with n=10 time intervals. Markov chain Monte Carlo (MCMC) sampling was performed for $20\times 10^{6}$ iterations, with 20% discarded as burn-in. Convergence of MCMC runs was assessed using Tracer (81), and posterior medians with 95% Highest Posterior Density (HPD) intervals were visualized in R.

We further applied a serial birth-death skyline model (BDSKY) (82) to estimate the effective reproductive number $R_{e}$ and the becoming uninfectious rate δ. The sampling proportion was also modeled as a piecewise-constant function As in the BSP model, 10 time intervals were used for piecewise constant estimates of model parameters.

We employed the HKY nucleotide substitution model with gamma-distributed rate variation among sites (HKY+Γ model) and a lognormal relaxed molecular clock. Given the narrow sequence sampling window resulting in a modest root-to-tip divergence correlation (*r*=0.24), we used a strongly informed prior on the molecular clock rate, with priors on the mean rate set to a lognormal distribution with a mean of $3.5\times 10^{-3}$ mutations/site/year and a standard deviation of $5.0\times 10^{-4}$. The standard deviation of the rate also followed a lognormal prior (mean = $5.0\times 10^{-4}$, standard deviation = $5.0\times 10^{-4}$), as suggested by previous studies (49, 83, 84). Different substitution models for BSP and BDSKY models were iused to demonstrate the robustness of conclusions and the consistency of results across methodological frameworks.

Prior distributions for the BDSKY model parameters were set similarly to values used in other studies of Eastern European countries (49):

• A uniform distribution for the date of origin, with an upper bound of 60 years and a lower bound of 19 years.
• A lognormal distribution for the effective reproduction number $R_{e}$ (mean = 1.0, standard deviation = 1.25), with an upper bound of 5.
• A lognormal distribution for the becoming noninfectious rate δ (mean = 0.0, standard deviation = 1.0 for normal distribution), constrained to the interval [0.05,6].
• A beta distribution for the sampling proportion (α=1.0,β=10.0), constrained to the interval [0,0.001]. These settings were informed by estimates from (49), given a comparable ratio of analyzed sequences to the estimated number of diagnosed PLHIV in both studies.
• A uniform distribution on the interval [0,1] for the freqParameter.
• An exponential distribution (mean = 1) for the gammaShape parameter.
• A lognormal distribution (mean = 1.0, standard deviation = 1.25) for the kappa parameter.

Markov chain Monte Carlo (MCMC) sampling was performed for $40\times 10^{7}$ iterations, with 20% discarded as burn-in and with convergence of MCMC assessed using Tracer (81). Effective reproduction numbers inferred using the BDSKY model were visualized with the R package bdskytools.

## 3 Results

### 3.1 Phylogenetic analysis of HIV transmission routes

The HIV transmission routes for Belarusian HIV sequences were analyzed at both international and within-country scales.

**Figure 1:**
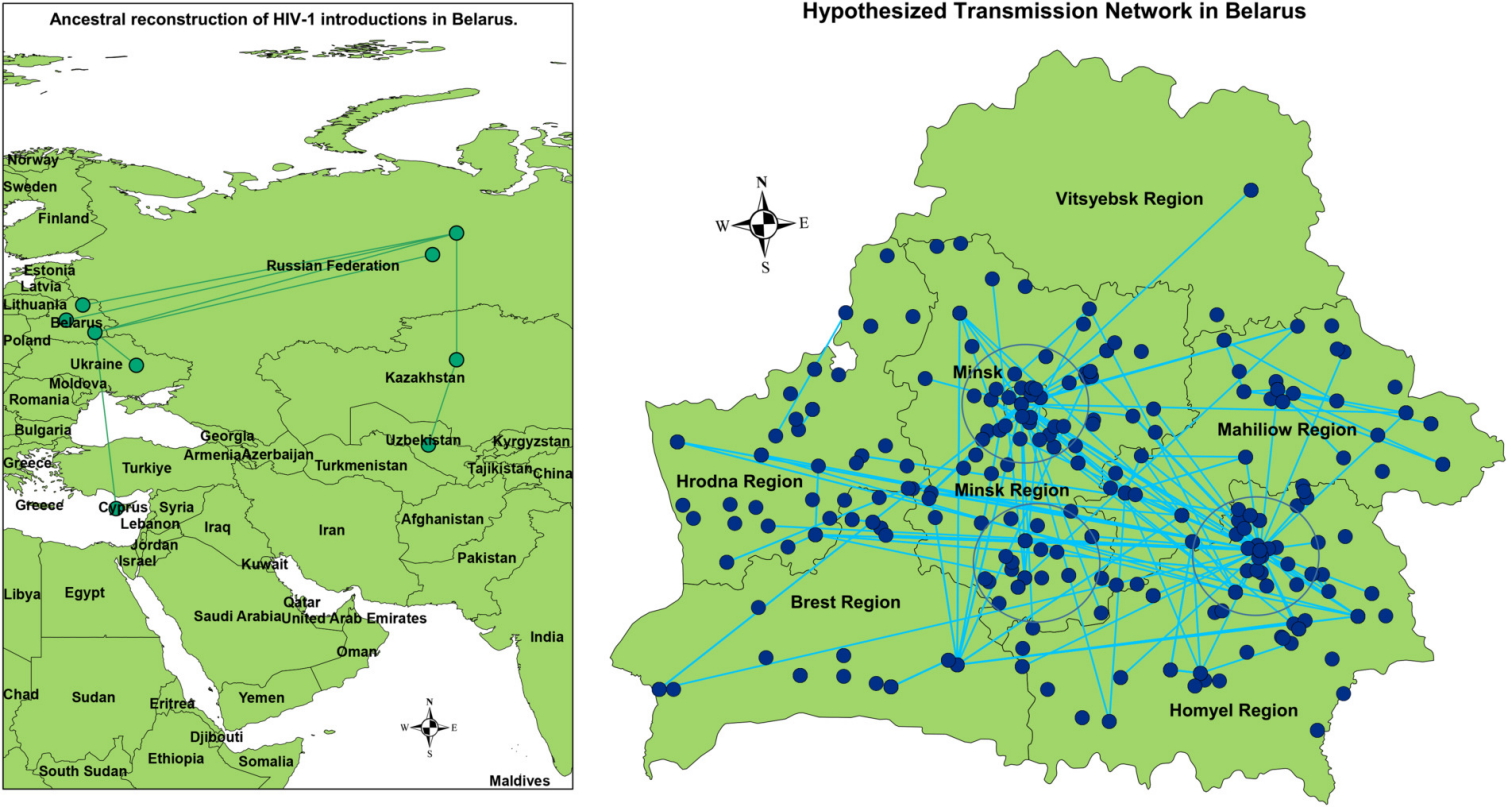
Left: Phylogenetically reconstructed between-country HIV-1 transmission network. Right: Inferred non-directional HIV intra-country transmission network. Largest transmission clusters are highlighted by circles. The displayed nodes may have multiplicity greater than one, but for simplicity, they are presented as single points.

In the international phylogeny, which included the analyzed Belarusian sequences alongside globally sampled sequences, Belarusian sequences generally clustered with sequences from other FSU countries, as expected. Ancestral phylogeographic reconstruction revealed multiple links to HIV lineages from Russia (average parent and child posterior probabilities for branches indicative of introductions: 0.94 (SD = 0.10), 0.98 (SD = 0.05), respectively), as well as connections with Ukraine [parent = 0.99 (SD = 0.00), child = 0.99 (SD = 0.00)] and Cyprus [parent = 0.82 (SD = 0.03), child = 0.90 (SD = 0.19)]. The latter is the only non-post-Soviet country with such links detected (see the left panel of Figure 1); the presence of 10 Belarusian sequences within the corresponding clade suggests a single introduction followed by limited intra-country transmission.

The vast majority of Belarusian sequences formed distinct clades, highlighting the major role of intra-country transmissions. More specifically, we identified six clusters that each have a Belarusian most recent common ancestor (MRCA), contain at least 30 nodes, and are composed of at least 85% Belarusian sequences. Collectively, these clusters represent approximately 73% of all Belarusian sequences, with one cluster alone accounting for 54% (see Supplementary Figure S3).

**Figure 2:**
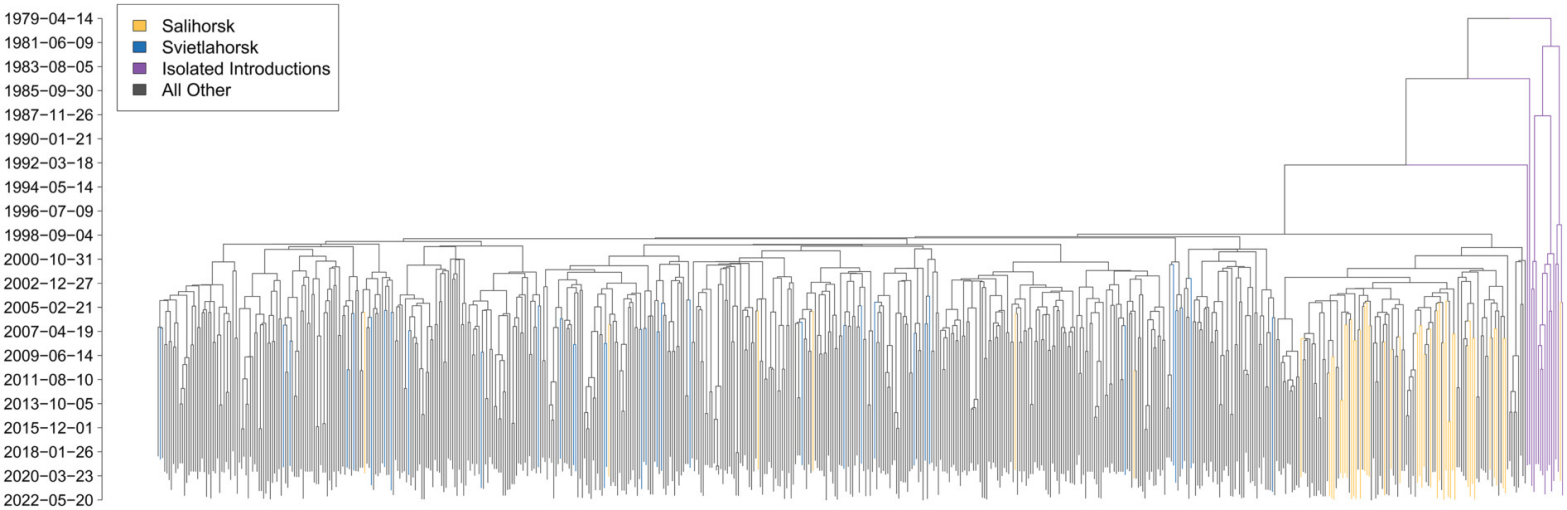
Timed phylogeny of the Belarusian HIV sequences. Clades containing sequences from Svietlahorsk (cluster 2) and Salihorsk (cluster 3), as well as those likely corresponding to isolated HIV introductions, are highlighted in different colors.

Most inferred potential transmission links were associated with the central (Minsk region) and south-eastern (Homyel region) parts of the country (see the right panel of Figures 1, 2) In particular, three large and highly interconnected clusters were identified, roughly corresponding to:

1. the metropolitan area of the capital Minsk,
2. the north-western part of the Homyel region, including the cities of Svietlahorsk, Zlobin, and Recyca,
3. the southern part of the Minsk region around the city of Salihorsk.

Clusters 2 and 3 correspond to industrial regions with a long history of HIV spread (10). In particular, Svietlahorsk and its surrounding area is considered a hotspot of the HIV epidemic and the location of the largest outbreaks identified in recent decades, most of which have been associated with high prevalence of injection drug use (85).

Significantly fewer within-country transmission links were associated with the western part of the country (Hrodna and Brest regions) compared to the eastern regions(see Figure 1). The main exceptions were a few links involving the city of Hrodna (capital of the Hrodna region) and the vicinity of the town of Luninets, which is adjacent to the Salihorsk cluster. This is consistent with their lower HIV prevalence, which is 1.8-fold and 2.4-fold below the national average, respectively.

The assortativity coefficient (a network parameter that quantifies the tendency of nodes with shared characteristics to connect—ranges from −1 (complete disassortative mixing) to 0 (random mixing) to 1 (perfect assortative mixing)), exhibited different behavior depending on the geographic level of aggregation.

At the regional level, the coefficient was relatively high (r=0.521), indicating that sequences from the same region were more likely to be linked than sequences from different regions. At the city level, the assortativity coefficient was moderate (r=0.345), suggesting that this association was much lower on the settlement level.

### 3.2 Phylodynamic inference of the effective reproductive number and effective population size

**Figure 3:**
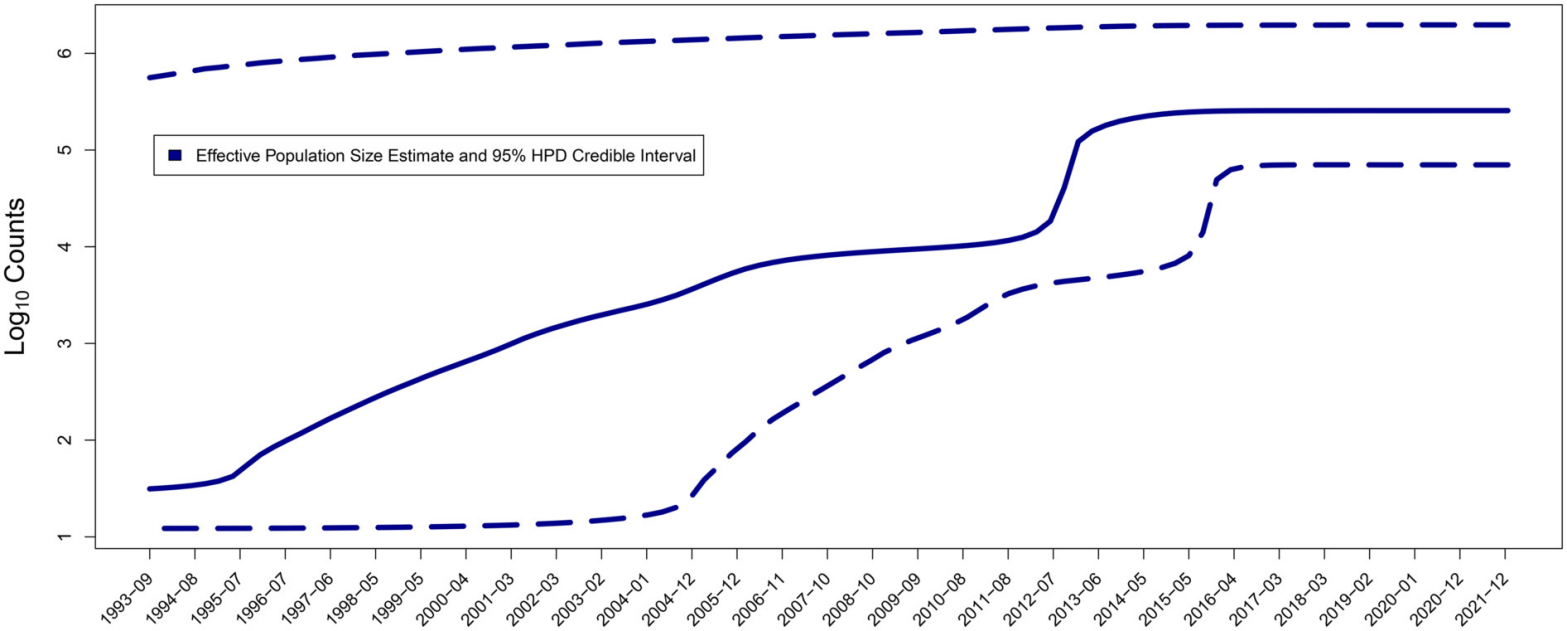
Median estimates and the corresponding 95% highest posterior density (HPD) intervals of the effective infected population size $N_{e}$.

**Figure 4:**
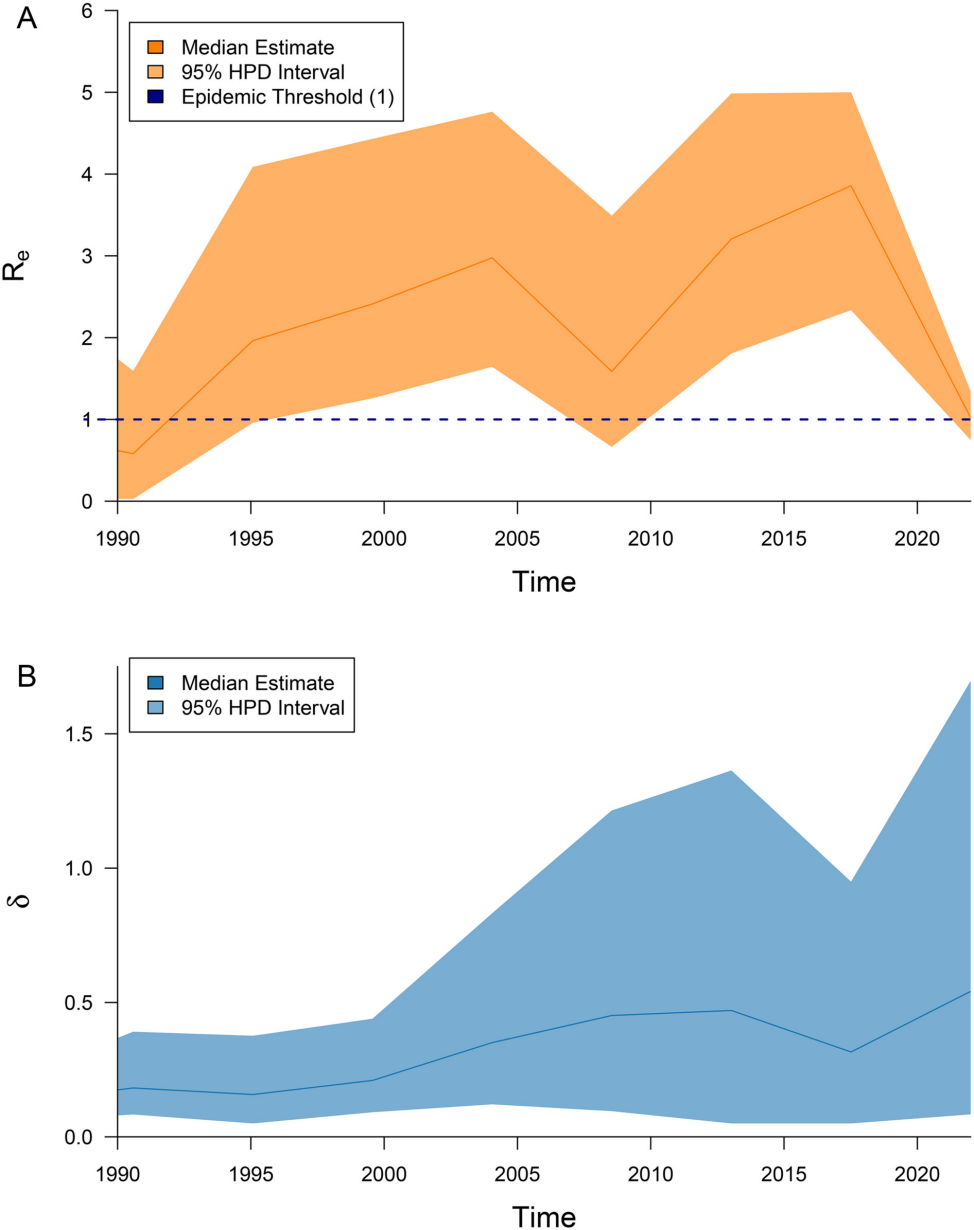
Median estimates and the corresponding 95% highest posterior density (HPD) intervals are presented in panel **(A)** for the effective reproduction number $R_{e}$ (orange), and in panel **(B)** for the becoming uninfectious rate δ (blue).

The median estimates of the effective population size, effective reproduction number, and becoming uninfectious rate, along with their 95% highest posterior density (HPD) intervals, are summarized in Figures 3, 4, and the timed phylogeny reconstructed using the Bayesian skyline model is shown in Figure 2.

In general, we observed a clear trend of epidemic growth from the onset of the epidemic through approximately the second half of the 2010s. During this period, the effective reproduction number ($R_{e}$) consistently remained above one, beginning in the early 1990s. After that, the trend appears to shift, with the effective population size ($N_{e}$) stabilizing and $R_{e}$ gradually approaching the epidemic threshold toward the end of the study period.

On a finer scale, the analysis revealed three identifiable stages of the HIV epidemic:

(A) the preliminary stage (roughly before the mid-90s);
(B) two epidemic waves: the first from the mid-1990s to mid-2000s, and the second during the 2010s.

The preliminary stage was likely driven mostly by external introductions. Its trace, along with that of later isolated introductions, may be reflected in the phylogeny by a small clade of HIV genotypes with most recent common ancestors dating to the 1980s [95% HPD for tMRCA: (39.8903–40.7611)], prior to the first reported HIV cases in the FSU countries (Figure 2).

The start of the first large wave (∼1997–2007, according to $N_{e}$ dynamics; Figure 3) aligns with the first large-scale HIV outbreak detected in the city of Svietlahorsk in mid-1996. Around this time, we observe the beginning of a gradual rise in the effective infected population size $N_{e}$ (Figure 3) and a burst in HIV genomic diversity (Figure 2).

The vast majority of sampled lineages coalesce to a most recent common ancestor around this period. Notably, sequences sampled in Svietlahorsk are distributed across much of the HIV phylogeny, whereas sequences from Salihorsk—despite comparable cluster size—generally form a distinct clade (Figure 2).

Overall, the first wave appears gradual and reflects a common pattern observed in post-Soviet countries, where the epidemic was driven by a combination of nosocomial transmission, sexual transmission, and injection drug use (86, 87). The second wave (∼2014–2016, based on $N_{e}$ dynamics; Figure 3) was more rapid and steep. It is possible that it corresponds to the spread of a new generation of injection drugs that significantly increased transmissions, that is well-documented for other Eastern European countries (88, 89).

Similar trends, though with slightly shifted time frames, are observed in the dynamics of the effective reproductive number $R_{e}$ (Figure 4). During the first wave, the estimated median ${\hat{R}}_{e}$ peaked at 2.98, closely matching the time window suggested by $N_{e}$ dynamics. For the second wave, the dynamics of $R_{e}$ peaked at ${\hat{R}}_{e}=3.86$ and points to an earlier start around 2009–2010.

Finally, although strong priors have been used for clock rate due to the narrow sequence sampling window, comparison of the prior and posterior distributions of clock rates (see Supplementary Figure S1) indicates that the posterior clock rate is data-driven.

The becoming uninfectious rate—i.e., the inverse of the average duration of the infectious period—underwent some fluctuations over time. However, the general trend was towards the rate increase, corresponding to a decrease in the average infectious period from approximately ∼7.7 years in the late 1980s to ∼1.9 years in the 2020s.

## 4 Discussion

This study presents findings on the HIV epidemiological dynamics in Belarus, inferred from genomic surveillance data. Eastern European and FSU countries continue to face significant challenges related to the HIV epidemic, while public health and social intervention efforts in the region are still developing—particularly when compared to Western Europe. At the same time, the epidemiological dynamics of HIV in this region remain poorly understood. This study contributes towards filling that knowledge gap and complements several recent studies on HIV transmission dynamics in other FSU countries (48, 49, 52).

The study revealed a complex history of HIV spread in Belarus since the start of the epidemic in 1987. In general, the estimates of the effective reproduction number $R_{e}$ remained above one throughout most of the analyzed period, indicating sustained transmission (90, 91), and, as discussed above, began to decline only after approximately 2018.

However, the dynamics of HIV transmission were not uniform during this time. The study identified three epidemic waves, separated by transitional periods. The first wave, roughly prior to 1996–1998, was characterized by only minimal growth in the effective infected population size ($N_{e}$), and was likely driven by relatively infrequent HIV introductions from abroad.

The next two waves—occurring from the mid-1990s through the early 2000s, and again during the early to late 2010s—can be linked to significant shifts in the epidemiological landscape. The second wave was likely triggered by a large-scale outbreak in the city of Svietlahorsk in mid-1996. This outbreak not only marked a turning point in national transmission dynamics, but also likely served as the source of most of the HIV genetic heterogeneity circulating in Belarus in the subsequent decades. This is supported by the observation that the vast majority of sampled lineages coalesce to a most recent common ancestor around this period, suggesting that the subsequent epidemic was largely driven by intra-country transmission of a single HIV lineage that rapidly diversified following the Svietlahorsk outbreak. Further evidence comes from the sequence distribution along the phylogeny, with sequences from Svietlahorsk being spread across much of the tree, in contrast with, for instance, sequences from Salihorsk–another HIV infection hotspot—that largely form a distinct clade. (Figure 2).

The third wave is likely associated with the introduction of a new generation of injection drugs and changes in injection practices, which greatly accelerated the pace and extent of HIV transmission (92, 93). Importantly, the dynamics of the effective reproductive number $R_{e}$ (Figure 4) suggests that this wave began several years before the epidemiologically confirmed events, indicating that the observed outbreaks were merely visible manifestations of underlying epidemiological processes that had already been underway and were not yet detected by traditional surveillance system. Such situations are not uncommon and have been observed in other countries—for example, during the HIV/HCV epidemic wave in the United States driven by opioid abuse at approximately the same time (93–95).

The periods of epidemic waves were interspersed with recession periods, which may be partially explained by public health interventions implemented during those periods. The drop in estimated values of $R_{e}$ around 2005 could reflect the delayed effects of multiple governmental efforts, including the UNAIDS intervention program launched in 1997 (10), as well as additional measures introduced in response to the initial outbreak. Similarly, the decline in the estimates of the effective reproduction number $R_{e}$ and infectious period observed since 2018 may be attributed to the implementation of a new clinical protocol, establishing antiretroviral therapy (ART) as a key component of healthcare for individuals living with HIV, and recommending ART for all PLHIV (96). Following this policy change, Belarus has made significant progress toward achieving the UNAIDS 95–95–95 targets (i.e., ensuring that 95% of people living with HIV know their status, 95% of those diagnosed receive antiretroviral therapy, and 95% of those on treatment achieve viral suppression). While in 2018 the percentages were estimated to be 77–58–37, by January 1, 2024, they had improved to 92.7–91.3–85.6 (97).

It is interesting to compare HIV dynamics in Belarus with the dynamics in neighboring Ukraine, also inferred using phylodynamic analysis (49). In particular, the general trend towards the becoming uninfectious rate increase (and, equivalently, decrease in the average infectious period) differs from the dynamics observed in northern Ukraine, where, as demonstrated in (49), the average infectious period has been increasing over time. At the same time, after 2013 the dynamics in Belarus show certain similarities to those observed in the Odessa region of Ukraine, where the estimated average infectious period decreased from ∼5 years in the 2000s to ∼1.1 years in 2013–2016, potentially due to the implementation of the transmission reduction intervention project in that region (49). Similarly, several harm reduction programs (such as needle exchange) were implemented in Belarus around the same time, followed by the introduction of the antiretroviral therapy protocol mentioned above. Thus, phylodynamic analyses both here and in (49) highlight the relative effectiveness of such programs in reducing HIV transmission among Eastern European populations.

The reconstruction of HIV introduction routes revealed linkages with neighboring Russia and Ukraine, which is to be expected, given the historically close economic and cultural ties between these regions, as well as largely unrestricted population movement across borders during most of the study period. The only detected link with a non–post-Soviet country was with Cyprus, which is plausibly explained by its longstanding popularity as a tourist destination for the Belarusian population in the decades preceding this study.

Within Belarus, the major regions contributing to HIV spread include the capital city of Minsk—as expected, given its role as a cultural, economic, and demographic hub as well as the Salihorsk and Svietlahorsk–Zlobin metropolitan areas in central and eastern Belarus, respectively where injection drug use has been endemic. This agrees with surveillance data, according to which the Homyel Region, that contains Svietlahorsk and Zlobin, accounts for nearly half of all HIV cases in the country. Both Salihorsk and Svietlahorsk–Zlobin are industrial regions with extensive mining and chemical industries, where limited social and recreational options may contribute to higher rates of HIV spread, in part due to elevated rates of intravenous drug use and associated high-risk behaviors.

Phylogenetically, however, the HIV subpopulations circulating in these regions are distinct (Figure 2) and may play different roles in the overall HIV transmission dynamics. In addition to the likelihood that Svyetlahorsk served as a key source of intra-country HIV diversity as discussed above, various geographical, economic, and environmental factors may also contribute towards the observed phylogenetic patterns. Svyetlahorsk is located along a major transport corridor connecting central and eastern Belarus and is surrounded by several towns of similar size, facilitating regional mobility. It has a legacy of Soviet-era industries, some of which have declined in recent decades, and is known for having one of the poorest ecological conditions in the country. These factors are associated with higher levels of out-migration and population movement, potentially contributing to both inward and outward HIV transmission. In contrast, Salihorsk is more geographically isolated and economically supported by the potash mining sector, which has contributed to population stability. These differences are consistent with the observed phylogenetic patterns: dispersed HIV lineages in Svyetlahorsk vs. more localized clustering in Salihorsk.

It is important to acknowledge that the findings presented in this study are subject to several limitations. First, the analysis covers the period only up to 2022, and therefore does not account for potential changes in epidemiological dynamics resulting from the demographic and social impacts of recent geopolitical events in the region. The analysis of such effects will only be possible once sufficient post-2022 data becomes available, and should be the focus of a separate, dedicated study. The same applies to the assessment of recently introduced HIV containment policies.

It should also be acknowledged that any analysis of HIV data may be influenced by under-reporting, delays in reporting due to the long incubation period of the virus (98), and sampling biases related to the social stigma surrounding HIV (99–101) and uneven sequencing capabilities among different regions. In particular, sequencing costs in non-Western countries remain high, and sequencing technologies are not yet as widely accessible to public health researchers as they are in Western Europe or North America. As pertains this study, only samples deemed to be of certain priority have been sequenced in Belarus and were thus available for this analysis. This may have introduced certain selection bias, as sequencing at the RCHEPH has been routinely performed only for patients with suspected drug resistance. Similarly, sampling biases could potentially have an impact on the global transmission analysis results.

As of September 2024, the Belarusian Ministry of Health and RCHEPH are actively working to expand sequencing capacity and increase funding to support comprehensive sequencing of all HIV samples from newly diagnosed individuals. This initiative is planned to be implemented in stages starting in 2025, and is expected to generate a larger dataset with more uniform coverage in the coming years. These expanded data will offer valuable insights into HIV dynamics in Eastern Europe, with the ultimate goal of enabling more targeted intervention strategies and optimizing resource allocation.

## Data Availability

Publicly available datasets were analyzed in this study. This data can be found here: The Belarusian HIV pol gene amplicons used in this study are publicly available on GenBank (accession numbers OP330328–OP331194).

## Supplementary material

Supplementary Data Sheet 1. Phylodynamics Analysis of HIV Epidemic History in Belarus in 1987-2022 by Kirpich et al.

## References

[1] unaidsorg. Data from: UNAIDS fact sheet (2024). Available online at: https://www.unaids.org/en/resources/fact-sheet (Accessed March 26, 2025).

[2] unaidsorg. Data from: UNAIDS 2023 report: chapter 1 (2023). Available online at: https://thepath.unaids.org/wp-content/themes/unaids2023/assets/files/2023_report_chapter_1.pdf (Accessed March 26, 2025).

[3] Carter A Zhang M Tram KH Walters MK Jahagirdar D Brewer ED. Content global, regional, and national burden of HIV/AIDS, 1990–2021, and forecasts to 2050, for 204 countries and territories: the Global Burden of Disease Study 2021. Lancet HIV. (2024) 11(12):e807–22. 10.1016/S2352-3018(24)00212-1 39608393 PMC11612058

[4] unaidsorg. Data from: UNAIDS global AIDS update 2024 (2024). Available online at: https://www.unaids.org/sites/default/files/media_asset/2024-unaids-global-aids-update-eeca_en.pdf (Accessed March 26, 2025).

[5] belstatgovby. Data from: Belarus population statistics–belstat.gov.by (2024). Available online at: https://www.belstat.gov.by/ofitsialnaya-statistika/solialnaya-sfera/naselenie-i-migratsiya/naselenie/ (Accessed March 26, 2025).

[6] Rytik PG Kucherov II Podolskaya IA Korzhev MO Glazovsky VA Firsova NP. Epidemiological analysis of HIV infection in the Republic of Belarus (In Russian). Med News. (1999) 3:3–7. https://www.mednovosti.by/journal.aspx?article=2554

[7] Eremin V Gasich E Eremin S Ambarcumian E Lukashov V. HIV/AIDS epidemic in Belarus. Retrovirology. (2010) 7:P112. 10.1186/1742-4690-7-S1-P112

[8] Eremin V Meleshko L Verevochkin S Zhdanovskaja O Gasich E Karamov E. XIV International AIDS Conference, Barcelona, 7-12 July 2002. Abstracts. (2002). p. 469. Abstract no. TuPeC4802.

[9] Eremin VF Karamov EV Lazouskaya NV Adema KW Gasich EL Baan E. Abstracts from the 10th European AIDS Conference (EACS). (2005). Abstract Book; Vol. 9 (PS1/5).

[10] Vickerman P Watts C. The impact of an HIV prevention intervention for injecting drug users in svetlogorsk, Belarus: model predictions. Int J Drug Policy. (2002) 13:149–64. 10.1016/S0955-3959(02)00071-3

[11] World Health Organization. Data from: HIV/AIDS treatment and care in Belarus: evaluation report (2014). Available online at: https://iris.who.int/handle/10665/350542 (Accessed March 26, 2025).

[12] Kachan VI Tkachova AI Gvozdeva E Urbanovich I Yakusik A Amico P. Resource flows and levels of spending for the response to HIV and AIDS in Belarus. BMC Res Notes. (2011) 4:1–6. 10.1186/1756-0500-4-248 21777473 PMC3156755

[13] UNAIDS. Data from: The National AIDS Spending Assessment (NASA) in Belarus 2008–2011 (2008). Available online at: https://www.unaids.org/sites/default/files/documents/belarus_2008-2011_en.pdf (Accessed March 26, 2025).

[14] Kolomiets ND Hanenko ON Fisenko EG Eremin VF Gasich EL Romanova ON. *Educational and Methodological Guide HIV Infection (Diagnosis, Epidemic Process in the Republic of Belarus)* (in Russian). Minsk: Belarusian Medical Academy of Postgraduate Education (BelMAO) (2013). Instruction for Use.

[15] Rusanovich AV Kolomiets ND Naroychik LK. Epidemiological situation of HIV/AIDS in the Republic of Belarus (in Russian). ARS Med. (2011) 14:361–2.

[16] UNAIDS. Data from: National AIDS spending assessment: a notebook on methods definitions and procedures to measure HIV and AIDS financial flows and expenditures at the country level (2009).

[17] Kolomiets ND Fisenko EG Naroychik LK Sergienko SV Rusanovich AV Tsarikova EV. Some trends in the development of the HIV/AIDS epidemic in the Republic of Belarus (in Russian). Medicine. (2014) 1(84):30–4.

[18] Our World in Data. Data from: Number of people living with HIV (2019). Available online at: https://ourworldindata.org/grapher/number-of-people-living-with-hiv (Accessed March 26, 2025).

[19] UNAIDS. Data from: UNAIDS global AIDS update 2024 (2024). Available online at: https://www.unaids.org/sites/default/files/media_asset/2024-unaids-global-aids-update-eeca_en.pdf (Accessed March 26, 2025).

[20] Rick F Odoke W Van Den Hombergh J Benzaken AS Avelino-Silva VI. Impact of coronavirus disease (COVID-19) on HIV testing and care provision across four continents. HIV Med. (2022) 23:169–77. 10.1111/hiv.13180 34632685 PMC8653012

[21] Viguerie A Song R Johnson AS Lyles CM Hernandez A Farnham PG. COVID-19-related excess missed HIV diagnoses in the United States in 2021. AIDS. (2024) 38:907–11. 10.1097/QAD.0000000000003829 38181069 PMC11249705

[22] Wójcik-Cichy K Piekarska A Jabłonowska E. Has COVID-19 changed the incidence and profile of late presenters for HIV infection in Lodz, Polish Reference Centre, Poland? J Clin Med. (2024) 13:4121. 10.3390/jcm13144121 39064161 PMC11278052

[23] UNAIDS. Data from: UNAIDS – Belarus (2024). Available online at: https://www.unaids.org/en/regionscountries/countries/belarus (Accessed March 26, 2025).

[24] UNAIDS. Data from: HIV estimates with uncertainty bounds (1990-present) (2024). Available online at: https://www.unaids.org/en/resources/documents/2024/HIV_estimates_with_uncertainty_bounds_1990-present (Accessed March 26, 2025).

[25] Kolomiets ND Klyuchareva AA Romanova ON Gasich EL Hanenko ON Klimovich NV. Modern view on diagnostics and monitoring of HIV infection in children (in Russian). Clin Infectiol Parasitol. (2018) 8:527–43. https://infecto.recipe.by/ru/?editions=2019-tom-8-n4

[26] World Health Organization. Data from: WHO validates elimination of mother-to-child transmission of HIV and Syphilis in Armenia, Belarus and the Republic of Moldova (2016). Available online at: https://www.who.int/news/item/08-06-2016-who-validates-countries-elimination-of-mother-to-child-transmission-of-hiv-and-syphilis (Accessed March 26, 2025).

[27] Pruneri G De Braud F Sapino A Aglietta M Vecchione A Giusti R. Next-generation sequencing in clinical practice: is it a cost-saving alternative to a single-gene testing approach? Pharmacoecon Open. (2021) 5:285–98. 10.1007/s41669-020-00249-0 33660227 PMC8160052

[28] Goodwin S McPherson JD McCombie WR. Coming of age: ten years of next-generation sequencing technologies. Nat Rev Genet. (2016) 17:333–51. 10.1038/nrg.2016.49 27184599 PMC10373632

[29] Knyazev S Hughes L Skums P Zelikovsky A. Epidemiological data analysis of viral quasispecies in the next-generation sequencing era. Brief Bioinform. (2021) 22:96–108. 10.1093/bib/bbaa101 32568371 PMC8485218

[30] Armstrong GL MacCannell DR Taylor J Carleton HA Neuhaus EB Bradbury RS. Pathogen genomics in public health. N Engl J Med. (2019) 381:2569–80. 10.1056/NEJMsr1813907 31881145 PMC7008580

[31] Preston BD Dougherty JP. Mechanisms of retroviral mutation. Trends Microbiol. (1996) 4:16–21. 10.1016/0966-842X(96)81500-9 8824790

[32] Roberts JD Bebenek K Kunkel TA. The accuracy of reverse transcriptase from HIV-1. Science. (1988) 242:1171–3. 10.1126/science.2460925 2460925

[33] Burke DS. Recombination in HIV: an important viral evolutionary strategy. Emerging Infect Dis. (1997) 3:253. 10.3201/eid0303.970301 PMC2627633 9284369

[34] Fraser C Lythgoe K Leventhal GE Shirreff G Hollingsworth TD Alizon S. Virulence and pathogenesis of HIV-1 infection: an evolutionary perspective. Science. (2014) 343:1243727. 10.1126/science.1243727 24653038 PMC5034889

[35] Sanjuán R Nebot MR Chirico N Mansky LM Belshaw R. Viral mutation rates. J Virol. (2010) 84:9733–48. 10.1128/jvi.00694-10 20660197 PMC2937809

[36] Abram ME Ferris AL Shao W Alvord WG Hughes SH. Nature, position, and frequency of mutations made in a single cycle of HIV-1 replication. J Virol. (2010) 84:9864–78. 10.1128/JVI.00915-10 20660205 PMC2937799

[37] Gasich E Bunas A Glinskaya I Skripko O Yurovsky P Kanonchyk K. Prevalence and correlates of pre-treatment HIV drug resistance among HIV-infected in the Republic of Belarus. In: *Abstracts Book of the 18th European Meeting on HIV & Hepatitis Treatment Strategies & Antiviral Drug Resistance*. (2020). Virtual Meeting; p. 87.

[38] Díez-Fuertes F Cabello M Thomson MM. Bayesian phylogeographic analyses clarify the origin of the HIV-1 subtype a variant circulating in former Soviet Union’s countries. Infect Genet Evol. (2015) 33:197–205. 10.1016/j.meegid.2015.05.003 25952568

[39] Désiré N Cerutti L Le Hingrat Q Perrier M Emler S Calvez V. Characterization update of HIV-1 m subtypes diversity and proposal for subtypes a and d sub-subtypes reclassification. Retrovirology. (2018) 15:1–7. 10.1186/s12977-018-0461-y 30577842 PMC6303845

[40] Abidi SH Aibekova L Davlidova S Amangeldiyeva A Foley B Ali S. Origin and evolution of HIV-1 subtype A6. PLoS One. (2021) 16:e0260604. 10.1371/journal.pone.0260604 34898626 PMC8668117

[41] Bobkov A Kazennova E Khanina T Bobkova M Selimova L Kravchenko A. An HIV type 1 subtype A strain of low genetic diversity continues to spread among injecting drug users in Russia: study of the new local outbreaks in Moscow and Irkutsk. AIDS Res Hum Retroviruses. (2001) 17:257–61. 10.1089/088922201750063188 11177409

[42] Lazouskaya NV Eremin VF Adema KW Gasich EL Baan E Lukashov VV. The HIV type 1 epidemic in Belarus: predominance of eastern European subtype a strains and circulation of subtype b viruses. AIDS Res Hum Retroviruses. (2005) 21:830–3. 10.1089/aid.2005.21.830 16218809

[43] Thomson MM De Parga EV Vinogradova A Sierra M Yakovlev A Rakhmanova A. New insights into the origin of the HIV type 1 subtype a epidemic in former Soviet Union’s countries derived from sequence analyses of preepidemically transmitted viruses. AIDS Res Hum Retroviruses. (2007) 23:1599–604. 10.1089/aid.2007.0166 18160020

[44] Eremin V Gasich E Sasinovich S Domnich M. HIV-1 subtypes in Belarus, 2009–2014. In: Minisymposium “The epidemiology of HIV-1, HCV & HBV in the Baltic region”; 2015 Mar 19–20. Stockholm: Karolinska Institutet (2015). p. 17.

[45] Eremin V Gasich E Fisenko E Yurovsky P Sasinovich S Domnich M. The HIV outbreak in Minsk. In: Caplinskas S, editor. Vilnius International Summit on Communicable Diseases; 2016 Jun 26–Jul 01. Vilnius: Centre for Communicable Disease and AIDS (ULAC) (2016). p. 64.

[46] Eremin V Gasich E Sasinovich S Domnich M. HIV-1 subtypes in Belarus, 2009–2016. J Virus Erad. (2016) 2(1).

[47] Volz EM Koelle K Bedford T. Viral phylodynamics. PLoS Comput Biol. (2013) 9:e1002947. 10.1371/journal.pcbi.1002947 23555203 PMC3605911

[48] Lebedev A Kuznetsova A Kim K Ozhmegova E Antonova A Kazennova E. Identifying HIV-1 transmission clusters in Uzbekistan through analysis of molecular surveillance data. Viruses. (2022) 14:1675. 10.3390/v14081675 36016298 PMC9413238

[49] Vasylyeva TI Zarebski A Smyrnov P Williams LD Korobchuk A Liulchuk M. Phylodynamics helps to evaluate the impact of an HIV prevention intervention. Viruses. (2020) 12:469. 10.3390/v12040469 32326127 PMC7232463

[50] Serwin K Scheibe K Urbanska A Aksak-Was B Karasinska-Cieslak M Zabek P. Phylodynamic evolution of HIV-1 A6 sub-subtype epidemics in Poland. J Med Virol. (2024) 96:e29482. 10.1002/jmv.29482 38381668

[51] van de Klundert MA Antonova A Di Teodoro G Ceña Diez R Chkhartishvili N Heger E. Molecular epidemiology of HIV-1 in eastern Europe and Russia. Viruses. (2022) 14:2099. 10.3390/v14102099 36298654 PMC9609922

[52] Aibekova L Bexeitova A Aldabergenova A Hortelano G Ge Z Yi F. Transmission of HIV and HCV within former Soviet Union countries. Can J Gastroenterol Hepatol. (2020) 2020:9701920. 10.1155/2020/9701920 32733822 PMC7378597

[53] Monakhov NE Ermakov AI Obizhaeva ES Vinogradova TN Lioznov DA. Molecular-genetic monitoring of circulating HIV-1 variants in St. Petersburg (in Russian). HIV Infect Immunosuppression. (2024) 16:106–17. 10.22328/2077-9828-2024-16-2-106-117

[54] Piterskiy M Gusev A Khodakov O Zakharova YA Semenov A. HIV-1 subtype diversity, phylogenetic analysis and study of drug resistance in strains circulating in the Ural Federal district (in Russian). J Microbiol Epidemiol Immunobiol. (2022) 99:38–53. 10.36233/0372-9311-178

[55] Maksimenko LV Totmenin AV Gashnikova MP Astakhova EM Skudarnov SE Ostapova TS. Genetic diversity of HIV-1 in krasnoyarsk krai: area with high levels of HIV-1 recombination in Russia. Biomed Res Int. (2020) 2020:9057541. 10.1155/2020/9057541 32964045 PMC7501552

[56] Brenner BG Ibanescu RI Osman N Cuadra-Foy E Oliveira M Chaillon A. The role of phylogenetics in unravelling patterns of HIV transmission towards epidemic control: the Quebec experience (2002–2020). Viruses. (2021) 13:1643. 10.3390/v13081643 34452506 PMC8402830

[57] Alexiev I Campbell EM Knyazev S Pan Y Grigorova L Dimitrova R. Molecular epidemiological analysis of the origin and transmission dynamics of the HIV-1 crf01_ae sub-epidemic in Bulgaria. Viruses. (2021) 13:116. 10.3390/v13010116 33467166 PMC7829743

[58] Bbosa N Ssemwanga D Nsubuga RN Kiwanuka N Bagaya BS Kitayimbwa JM. Phylogenetic networks and parameters inferred from HIV nucleotide sequences of high-risk and general population groups in Uganda: implications for epidemic control. Viruses. (2021) 13:970. 10.3390/v13060970 34073846 PMC8225143

[59] Thompson JD Higgins DG Gibson TJ. Clustal w: improving the sensitivity of progressive multiple sequence alignment through sequence weighting, position-specific gap penalties and weight matrix choice. Nucleic Acids Res. (1994) 22:4673–80. 10.1093/nar/22.22.4673 7984417 PMC308517

[60] Katoh K Standley DM. Mafft multiple sequence alignment software version 7: improvements in performance and usability. Mol Biol Evol. (2013) 30:772–80. 10.1093/molbev/mst010 23329690 PMC3603318

[61] Madeira F Pearce M Tivey AR Basutkar P Lee J Edbali O. Search and sequence analysis tools services from EMBL-EBI in 2022. Nucleic Acids Res. (2022) 50:W276–9. 10.1093/nar/gkac240 35412617 PMC9252731

[62] Struck D Lawyer G Ternes AM Schmit JC Bercoff DP. Comet: adaptive context-based modeling for ultrafast HIV-1 subtype identification. Nucleic Acids Res. (2014) 42:e144. 10.1093/nar/gku739 25120265 PMC4191385

[63] Pineda-Peña AC Faria NR Imbrechts S Libin P Abecasis AB Deforche K. Automated subtyping of HIV-1 genetic sequences for clinical and surveillance purposes: performance evaluation of the new rega version 3 and seven other tools. Infect Genet Evol. (2013) 19:337–48. 10.1016/j.meegid.2013.04.032 23660484

[64] Lole KS Bollinger RC Paranjape RS Gadkari D Kulkarni SS Novak NG. Full-length human immunodeficiency virus type 1 genomes from subtype c-infected seroconverters in India, with evidence of intersubtype recombination. J Virol. (1999) 73:152–60. 10.1128/JVI.73.1.152-160.1999 9847317 PMC103818

[65] Hadfield J Megill C Bell SM Huddleston J Potter B Callender C. Nextstrain: real-time tracking of pathogen evolution. Bioinformatics. (2018) 34:4121–3. 10.1093/bioinformatics/bty407 29790939 PMC6247931

[66] Huddleston J Hadfield J Sibley TR Lee J Fay K Ilcisin M. Augur: a bioinformatics toolkit for phylogenetic analyses of human pathogens. J Open Source Software. (2021) 6:2906. 10.21105/joss.02906 PMC8237802 34189396

[67] Stamatakis A. Raxml version 8: a tool for phylogenetic analysis and post-analysis of large phylogenies. Bioinformatics. (2014) 30:1312–3. 10.1093/bioinformatics/btu033 24451623 PMC3998144

[68] Sagulenko P Puller V Neher RA. Treetime: maximum-likelihood phylodynamic analysis. Virus Evol. (2018) 4:vex042. 10.1093/ve/vex042 29340210 PMC5758920

[69] Ishikawa SA Zhukova A Iwasaki W Gascuel O. A fast likelihood method to reconstruct and visualize ancestral scenarios. Mol Biol Evol. (2019) 36:2069–85. 10.1093/molbev/msz131 31127303 PMC6735705

[70] Felsenstein J. Evolutionary trees from dna sequences: a maximum likelihood approach. J Mol Evol. (1981) 17:368–76. 10.1007/BF01734359 7288891

[71] Campbell EM Boyles A Shankar A Kim J Knyazev S Cintron R. Microbetrace: retooling molecular epidemiology for rapid public health response. PLoS Comput Biol. (2021) 17:e1009300. 10.1371/journal.pcbi.1009300 34492010 PMC8491948

[72] Wertheim JO Leigh Brown AJ Hepler NL Mehta SR Richman DD Smith DM. The global transmission network of HIV-1. J Infect Dis. (2014) 209:304–13. 10.1093/infdis/jit524 24151309 PMC3873788

[73] Hassan AS Pybus OG Sanders EJ Albert J Esbjörnsson J. Defining HIV-1 transmission clusters based on sequence data. AIDS. (2017) 31:1211–22. 10.1097/QAD.0000000000001470 28353537 PMC5482559

[74] Novitsky V Steingrimsson JA Howison M Gillani FS Li Y Manne A. Empirical comparison of analytical approaches for identifying molecular HIV-1 clusters. Sci Rep. (2020) 10:18547. 10.1038/s41598-020-75560-1 33122765 PMC7596705

[75] Knyazev S. Data from: attribute_assortativity script github repository (2019). Available online at: https://github.com/Sergey-Knyazev/attribute_assortativity (Accessed March 26, 2025).

[76] Alexiev I Campbell EM Knyazev S Pan Y Grigorova L Dimitrova R. Molecular epidemiology of the HIV-1 subtype b sub-epidemic in Bulgaria. Viruses. (2020) 12:441. 10.3390/v12040441 32295123 PMC7232140

[77] Bouckaert R Heled J Kühnert D Vaughan T Wu CH Xie D. Beast 2: a software platform for bayesian evolutionary analysis. PLoS Comput Biol. (2014) 10:e1003537. 10.1371/journal.pcbi.1003537 24722319 PMC3985171

[78] Ayres DL Darling A Zwickl DJ Beerli P Holder MT Lewis PO. Beagle: an application programming interface and high-performance computing library for statistical phylogenetics. Syst Biol. (2012) 61:170–3. 10.1093/sysbio/syr100 21963610 PMC3243739

[79] Rambaut A Lam TT Max Carvalho L Pybus OG. Exploring the temporal structure of heterochronous sequences using tempest (formerly path-o-gen). Virus Evol. (2016) 2:vew007. 10.1093/ve/vew007 27774300 PMC4989882

[80] Drummond AJ Rambaut A Shapiro B Pybus OG. Bayesian coalescent inference of past population dynamics from molecular sequences. Mol Biol Evol. (2005) 22:1185–92. 10.1093/molbev/msi103 15703244

[81] Rambaut A Drummond AJ Xie D Baele G Suchard MA. Posterior summarization in bayesian phylogenetics using tracer 1.7. Syst Biol. (2018) 67:901–4. 10.1093/sysbio/syy032 29718447 PMC6101584

[82] Stadler T Kühnert D Bonhoeffer S Drummond AJ. Birth–death skyline plot reveals temporal changes of epidemic spread in HIV and hepatitis c virus (HCV). Proc Natl Acad Sci. (2013) 110:228–33. 10.1073/pnas.1207965110 23248286 PMC3538216

[83] Abecasis AB Vandamme AM Lemey P. Quantifying differences in the tempo of human immunodeficiency virus type 1 subtype evolution. J Virol. (2009) 83:12917–24. 10.1128/JVI.01022-09 19793809 PMC2786833

[84] Patiño-Galindo JÁ Gonzalez-Candelas F. The substitution rate of HIV-1 subtypes: a genomic approach. Virus Evol. (2017) 3:vex029. 10.1093/ve/vex029 29942652 PMC6007745

[85] Eremin VF Gasich EL Suetnov ON Grushko TP Grushko PN Sosinovich SV. Specifics of the development of the HIV/AIDS epidemic in the Homyel region in 2008–2011. Report 3. Zdravookhranenie. (2013) 4:14–26.

[86] UNAIDS/WHO Working Group on Global HIV/AIDS and STI Surveillance. UNAIDS/WHO epidemiological fact sheet – 2004 update: Belarus (Tech. Rep.). Joint United Nations Programme on HIV/AIDS (UNAIDS) and World Health Organization (WHO) (2004).

[87] Atlani L Caraël M Brunet JB Frasca T Chaika N. Social change and HIV in the former USSR: the making of a new epidemic. Social Sci Med. (2000) 50:1547–56. 10.1016/S0277-9536(99)00464-5 10795962

[88] Tarján A Dudás M Gyarmathy VA Rusvai E Tresó B. Csohán Á. Emerging risks due to new injecting patterns in hungary during austerity times. Substance Use Misuse. (2015) 50:848–58. 10.3109/10826084.2015.978672 25775136

[89] Botescu A Abagiu A Mardarescu M Ursan M. HIV/AIDS among injecting drug users in Romania. In: *Report of a Recent Outbreak and Initial Response Policies*. (2012). p. 5–14. https://idpc.net/publications/2012/11/hiv-aids-among-injecting-drug-users-in-romania https://cdn.sanity.io/files/6u5teakk/production/4a6aa5b1bbe2046e65cf664e04155c6d5ff43884.pdf?dl= (Accessed March 26, 2025).

[90] Nishiura H Chowell G. The effective reproduction number as a prelude to statistical estimation of time-dependent epidemic trends. In: Chowell G Hyman JM Bettencourt LMA Castillo-Chavez C, editors. Mathematical and Statistical Estimation Approaches in Epidemiology. Dordrecht: Springer (2009) 103–21. 10.1007/978-90-481-2313-1_5

[91] Delamater PL Street EJ Leslie TF Yang YT Jacobsen KH. Complexity of the basic reproduction number (r0). Emerging Infect Dis. (2019) 25:1. 10.3201/eid2501.171901 PMC6302597 30560777

[92] Des Jarlais DC Carrieri P. HIV infection among persons who inject drugs: ending old epidemics and addressing new outbreaks: authors’ reply. AIDS. (2016) 30:1858–9. 10.1097/QAD.0000000000001117 27351930 PMC4928690

[93] Peters PJ Pontones P Hoover KW Patel MR Galang RR Shields J. HIV infection linked to injection use of oxymorphone in Indiana, 2014–2015. N Engl J Med. (2016) 375:229–39. 10.1056/NEJMoa1515195 27468059

[94] Campbell EM Jia H Shankar A Hanson D Luo W Masciotra S. Detailed transmission network analysis of a large opiate-driven outbreak of HIV infection in the United States. J Infect Dis. (2017) 216:1053–62. 10.1093/infdis/jix307 29029156 PMC5853229

[95] Ramachandran S Thai H Forbi JC Galang RR Dimitrova Z Xia G A large HCV transmission network enabled a fast-growing HIV outbreak in rural Indiana, 2015. EBioMedicine. (2018) 37:374–81. 10.1016/j.ebiom.2018.10.007 30448155 PMC6284413

[96] National Legal Internet Portal of the Republic of Belarus. Data from: Approval of the clinical protocol diagnosis and treatment of patients with HIV infection (in Russian) (2021). Available online at: https://pravo.by/document/?guid=3961&p0=W21732132p (Accessed March 26, 2025).

[97] UNAIDS. Data from: Data (2024). Available online at: https://dsd.unaids.org/ (Accessed March 26, 2025).

[98] Yoshimura K. Current status of HIV/AIDS in the ART era. J Infect Chemother. (2017) 23:12–6. 10.1016/j.jiac.2016.10.002 27825722

[99] Brent RJ. The value of reducing HIV stigma. Social Sci Med. (2016) 151:233–40. 10.1016/j.socscimed.2016.01.014 26820574

[100] Marellapudi A Hussen SA Brown DN Fletcher MR Henkhaus ME Jones MD. Understanding and addressing privacy and confidentiality concerns associated with the provision of mobile HIV care: a qualitative study. AIDS Care. (2022) 34:575–9. 10.1080/09540121.2021.1921104 33938335 PMC8563507

[101] Kontomanolis EN Michalopoulos S Gkasdaris G Fasoulakis Z. The social stigma of HIV–AIDS: society’s role. HIV/AIDS-Res Palliat Care. (2017) 9:111–8. 10.2147/HIV.S129992 PMC5490433 28694709

