# Supplementary material for: Phylodynamics analysis of HIV epidemic history in Belarus in 1987–2022
Source: Front Epidemiol. 2025 Jul 21;5:1601976. doi: 10.3389/fepid.2025.1601976 (PMC12318975; doi:10.3389/fepid.2025.1601976)
Supplement: Supplementary Data Sheet 1. — Phylodynamics Analysis of HIV Epidemic History in Belarus in 1987-2022 by Kirpich et al. [file Datasheet1.pdf]

Supplementary Material: Phylodynamics Analysis of HIV Epidemic History in Belarus in 1987-2022

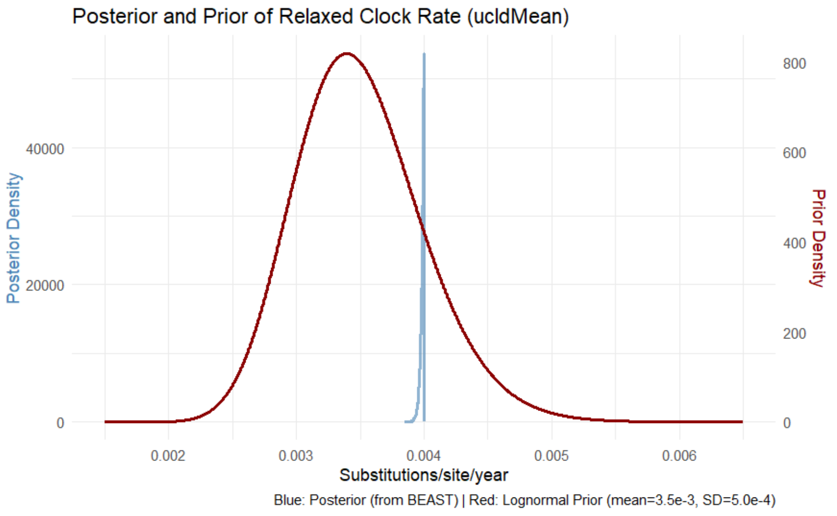

Figure S1. Prior (red) and posterior (blue) distributions of relaxed clock rate for BDSKY model.

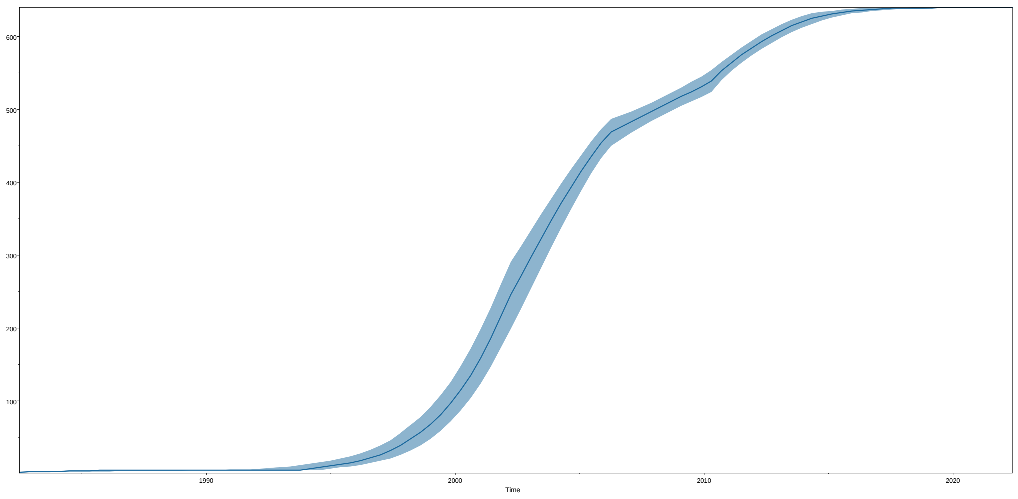

Figure S2. Lineages-through-time for BDSKY model. X-axis: calendar time. Y-axis: number of lineages in the viral phylogeny with living descendants at each time point.

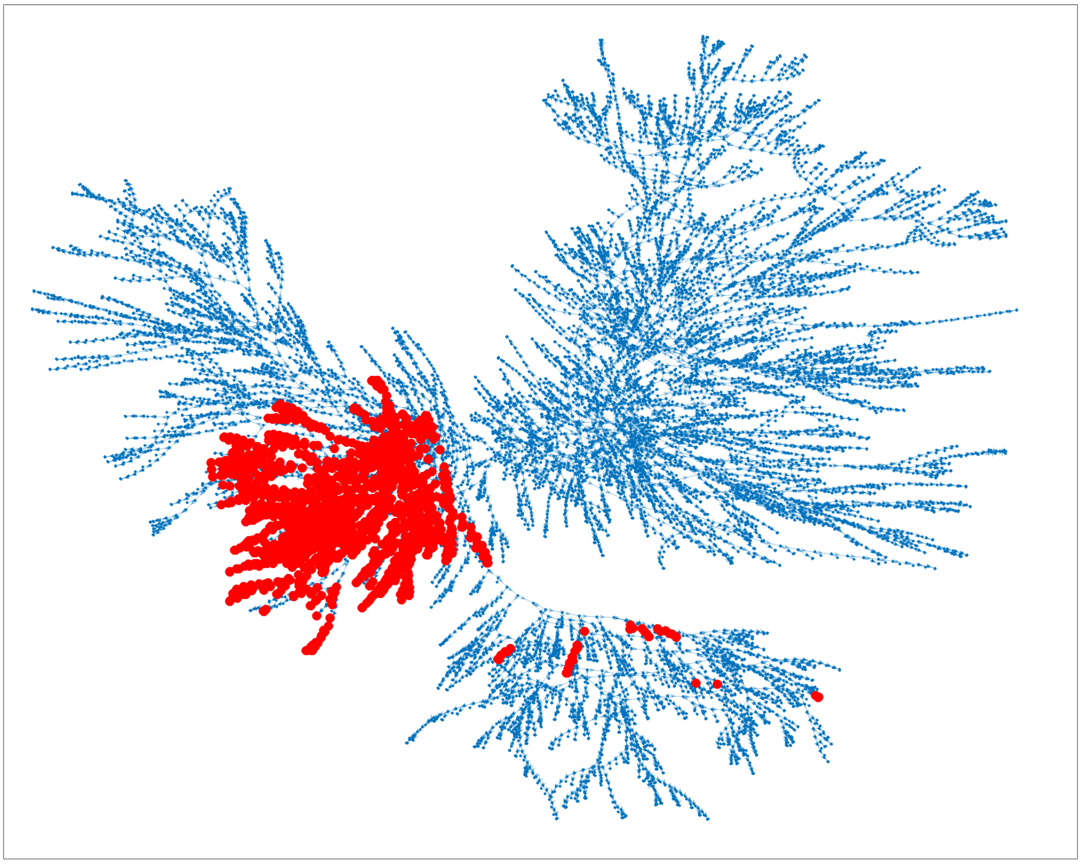

Figure S3. Global tree of HIV sequences. Belarusian sequences are highlighted in red.
